# Supplementary material for: Understanding the European energy crisis through structural causal models
Source: Nat Commun. 2026 Jul 17;17:6539. doi: 10.1038/s41467-026-75433-7 (PMC13379379; doi:10.1038/s41467-026-75433-7)
Supplement: Supplementary file 1 — Supplementary Information [file 41467_2026_75433_MOESM1_ESM.pdf]

Supplementary Information for
Understanding the European energy crisis through structural causal models

Sarah Schreyer,[1,2,*] Anton Tausendfreund,[1,2,*] Florian Immig,[3,*] Ulrich Oberhofer,[3,*] Julius Trebbien,[1,2] Aaron Praktiknjo,[4,5] Benjamin Schäfer,[3,†] and Dirk Witthaut[1,2,5,‡]

[1] *Institute of Climate and Energy Systems: Energy Systems Engineering (ICE-1), Forschungszentrum Jülich, 52428 Jülich, Germany*
[2] *Institute for Theoretical Physics, University of Cologne, 50937 Köln, Germany*
[3] *Institute for Automation and Applied Informatics, Karlsruhe Institute of Technology, 76344 Eggenstein-Leopoldshafen, Germany*
[4] *Chair for Energy System Economics, Institute for Future Energy Consumer Needs and Behavior (FCN), E.ON Energy Research Center, RWTH Aachen University, 52074 Aachen, Germany*
[5] *JARA-ENERGY, 52074 Aachen, Germany*

(Dated: June 10, 2026)

Supplementary Notes 2
Supplementary Note 1: Seasonality of nuclear availability, load and energy prices 2
Supplementary Note 2: Evaluation of SCMs 2
Supplementary Note 3: Structural coefficients of SCMs 4
Supplementary Note 4: Share of natural gas generation in french total generation 4
Supplementary Note 5: Price differences between France and its neighbours 4
Supplementary Note 6: Performance of GBT models 5
Supplementary Note 7: Further Shapley Flow results 5

Supplementary Methods 7
Supplementary Methods 1: Data sources and pre-processing 7

Supplementary Tables 9

Supplementary Figures 10

Supplementary References 21

SUPPLEMENTARY NOTES

Supplementary Note 1: Seasonality of nuclear availability, load and energy prices

Essential variables in the French electricity system display a strong seasonality as shown in Supplementary Fig. **1**. The river flow shows strong seasonal patterns. The demand for electricity is usually higher in the winter due to the prevalence of electric heaters [1]. Electricity market prices are determined by the balance of supply and demand, such that the price generally increases with the demand. In fact, we observe that prices are usually higher in winter than in summer.

The French electricity market heavily relies on nuclear power plants that require revisions. The demand and price are typically lowest during the summer and so are opportunity costs for the unavailability of nuclear power plants. Indeed we observe a pronounced seasonality of nuclear availability which closely follows the seasonality of the load (Supplementary Fig. **1**).

Seasonal affects can induce confounding. Typically, both the nuclear availability and the electricity market price are highest in winter as argued above. This can lead to a perceived violation of economic laws if one only considers the supply side and neglects the seasonal variation in demand.

Supplementary Note 2: Evaluation of SCMs

DoWhy [2, 3] checks how many local Markov conditions (LMC) are violated in a given graph using conditional independence (CI) tests. During CI tests, the conditional independence of all non-descendants of each node given its parents is checked [4].

Using LMCs, wrong graphs can be rejected, but a graph can not be declared as true with certainty. A benchmark is required of how many LMC violations are acceptable, above which graphs are rejected. To get a benchmark, we randomly permute the nodes of a given graph and test for violations of LMCs. The p-value, denoted as p_{LMC}, is defined as the probability of a randomly permuted graph to have fewer LMC violations than the original graph. We accept a given graph if the p-value falls below some threshold; otherwise it is rejected. Graphs of the same Markov equivalence class (MEC) have the same conditional independencies [5]. Thus, if too many of the permuted graphs lie in the same MEC, the conditional independence tests are not informative of the graph's correctness. A second p-value p_{MEC} is defined as the probability of a randomly permuted graph to lie in the same MEC as the given graph. The graph is considered falsifiable if the p-value is below a certain threshold. We use a significance level of 0.05 for both p-values. In conclusion, we say a graph is falsifiable if no more than 5 % of the randomly permuted graphs lie in the same MEC as the given graph. We say a graph is falsified if more than 5 % of the randomly permuted graphs have as few or fewer LMC violations as the given graph.

For all models (electricity market prices of France and Spain and net exports of France),

the p-value p_{MEC} is zero for 50 random node permuted graphs. None of the randomly permuted graphs lie in the same MEC as the given graphs. Therefore, we conclude that the corresponding causal graphs are falsifiable. To falsify the models, the violation of LMCs are compared against 50 random node permuted graphs. Out of the 352 possible LMC violations in case of the French model, the one for the electricity market prices violates 219 and the one for the net exports 222. The model for Spanish electricity market prices violates 171 out of 298 possible LMC violations. Nevertheless, the p-value p_{LMC} is zero for all models. Therefore, none of the permuted graphs violate as few or fewer LMCs than the original graph. The causal graph thus performs significantly better than random and we accept it.

We use the R^2 score to evaluate goodness of the fit for every structural equation. The R^2 score is defined as

$$R^2 = 1 - \frac{RSS}{TSS}. \tag{1}$$

Hereby, the total sum of squares TSS is the squared difference between observed values and their mean. The residual sum of squares RSS is the squared difference between model prediction and observed values. The ratio of RSS and TSS thus yields the fraction of variance that is not explained by the model. The R^2 score thus is the proportion of variance that the model can explain [6]. To calculate the R^2 value the data is split into 80% training data and 20% test data. The split is performed in weeks to minimise over fitting.

Different effects can lead to low R^2 scores. The used linear model might not be appropriate in case of strongly non-linear causal effects. Further, relevant variables might be missing which are collectively represented in the noise term in the structural equations. This can lead to the noise term dominating in the structural equation. This mechanism is especially prevalent in highly complex systems featuring many variables and dependencies which can not all be considered in the model. As the electricity market is such a highly complex system, we need to decide which dependencies the model should include and for which variables we accept that they are mostly influenced by noise terms.

The R^2 scores of all fits are depicted in Supplementary Fig. **3**. As our analysis focuses on the final targets, the electricity market prices and the net exports, their corresponding results are most important. We find a R^2 score of 0.92 for the electricity market price of France, 0.91 for the electricity market price of Spain and 0.83 for net exports of France. Hence, the proportion of variation in the target variables that the model can explain is high. The choice of a linear model, as well as the causal graph, appear to be a reasonable approximation.

All variables relevant to assess model evaluation, the R^2 scores and other metrics of the targets as well as the LMC violations, are listed in Supplementary Tab. **1**. The R^2 score of the nuclear availability of France is with 0.66 substantially lower than the R^2 scores of the target variables. This is to be expected since many influencing factors are not included in the model. For example, unforeseen corrosion damage lead to many reactors going into revision in 2021 and 2022 [7]. These variables are not included in the model.

Supplementary Note 3: Structural coefficients of SCMs

The raw structural coefficients of the SCM models are shown in Supplementary Fig. **2**. It is visible that French and Spanish Nuclear availability have similar impact on the French and Spanish electricity market prices. The units are mixed, for the electricity market prices units are given by $MWh \cdot tCO2e$ for Carbon price, unit less for gas price, $EUR/MWh \cdot GWh^{-1}$ and in $EUR/MWh \cdot GW^{-1}$ for the rest. For the net exports, units are given by $100\,tCO2e \cdot MW/EUR$ for the Carbon price, $100\,MW^2h/EUR$ for the gas price, $1/h$ for filling rate and unit less for the rest.

Supplementary Note 4: Share of natural gas generation in french total generation

France relies mostly on nuclear power for its electricity generation. Compared to its neighbours, gas generation plays a lesser role in French electricity production. In Supplementary Fig. **5** the share of gas generation of the total french generation is shown. During the energy crisis in the year 2022, an increase of share of gas generation can be observed.

Supplementary Note 5: Price differences between France and its neighbours

We use SCMs shown to analyse why French electricity market prices strongly increased during the energy crisis even though France relies less on gas than its neighbours. We conclude that two factors caused the sharp rise in French electricity market prices: The unavailability of French nuclear power plants leads to France being more dependent on its neighbours. This leads to higher French electricity market prices during the energy crisis when compared with its neighbours [8].

We analyse the difference of electricity market prices in France with respect to the neighbouring bidding zones in Supplementary Fig. **6**. Before the energy crisis, price differences with respect to Spain (ES), Belgium (BE) and Germany-Luxembourg (DE-LU) are generally small. They are mostly negative for ES and vary between positive and negative values for BE and DE-LU. During the crisis, price differences with respect to these neighbours increase sharply. Positive values are much more frequent than negative values, indicating imports from the respective bidding zones to France. Hence, the French electricity market could not isolate itself from its neighbours, which have a substantial share of natural gas in the electricity mix.

The situation is different for the bidding zone IT-North. Italy frequently imports electric power from its neighbours [9] and often sees comparably high electricity market prices. Accordingly, price differences between France and IT-North are often negative. During the energy crisis, the monthly average of the price difference remained negative while the standard deviation increased strongly.

Overall, the correlations of electricity market prices with Italy increased during the energy crisis [9] contrary to the other neighbours. That is, electricity market prices in France showed similar patterns it Italy, where natural gas provides contributes substantially to electricity generation (see main text).

Supplementary Note 6: Performance of GBT models

We complement the usage of SCMs with GBTs, as these can model non-linear patterns in the data. Hence, we also observe a better performance of the GBT models compared to the SCM models. We report R^2 scores and other metrics in Supplementary Table **1** and Supplementary Fig. **3** for the SCM and in Supplementary Table **2** and Supplementary Fig. **4** for the GBT model. Due to the non-linearity of GBT models, it is possible to substantially increase the R^2 score and thus the explained variance, particularly for features that cannot be adequately explained by linear SCMs. While all targets achieve an R^2 score of at least 92 percent, all non-root nodes in the causal graph have an R^2 score of at least 50 percent, and the majority have a score of over 70 percent. This confirms that GBT models are useful for analysing indirect effects in causal graphs and complement the results of SCMs in a meaningful way.

Supplementary Note 7: Further Shapley Flow results

Here we provide additional results for Shapley Flow in the GBT model. This analysis complements the results of the models of the electricity market prices and net exports of France in the main text.

An overview of the Shapley Flow values in the model of the electricity market price in Spain is provided in Supplementary Fig. **7**. As in the linear SCM, the gas price is the most important feature, complemented by the day-ahead wind and solar generation. These two features have larger impact for the Spanish electricity market prices than in the model for the electricity market prices in France. However, the type of correlation between the feature values pointing to the target and the Shapley flow values of the corresponding edges mostly resembles that of the French model.

We continue with a analysis of linear and non-linear effects of meteorological and economical features for the electricity market price in Spain. When analysing the direct effects of day-ahead load, ROR generation, and nuclear availability, we see similar effects on electricity market price in Spain as in France, see Supplementary Fig. **8**a-c. With regard to the indirect effects of air temperature, we see similar effects on the price via the day-ahead load as in France, up to a temperature of 20 °C in panel d. Above 20 °C, we see a more pronounced increase, which is probably due to increased use of air conditioning in Spain, compared to France.

In addition to the most relevant graph edges with respect to the Shapley Flow values, as discussed in the main text and in Supplementary Fig. **7**, we show for each of the three models a sub-graph with all nodes and edges (and thereby all flows). Here we focus on the temperature impact, specifically the indirect effects of air temperature, river temperature, and river flow rate on the model target, see Supplementary Fig. **9**. We note that each of these three features has a substantial impact on prices and net exports via both nuclear availability and run-of-river generation.

While the indirect effects of river flow rate on ROR generation are similar for all three models, the impact via nuclear availability in Spain is low in comparison.

To model the impact of hypothetical gas prices in Spain and nuclear availability in France during the energy crisis, we use a what-if scenario in which we apply the trained GBT models for predictions with hypothetical input values. In Supplementary Fig. **10**, we see that the Shapley flow values for both hypothetical gas prices as well as nuclear availability are in a similar range to the values for empirical values from the model's test set. Hence, we are not prone to extrapolate to completely unknown feature values. We conclude that the predictive power of the models for hypothetical gas prices and nuclear availability is reliable.

SUPPLEMENTARY METHODS

Supplementary Methods 1: Data sources and pre-processing

Data is obtained and processed as explained in the main text. Here we provide additional information.

Multiple bidding zone changes occurred during the period of investigation. Austria left a shared bidding zone with Germany and Luxembourg in 2018. The time series data of the bidding zone DE-AT-LU (until 2018-09-30) are therefore combined with the one of DE-LU (from 2018-10-01 onwards). The Italian bidding zones were modified on 2019-01-01 and on 2021-01-01 [10]. The production hubs IT-Brindisi, IT-Foggia and IT-Priolo were removed on 2019-01-01. On 2021-01-01, the production hub IT-Rossano was removed and the bidding zone IT-Calabria added. Meanwhile the region Umbria moved from IT-Centre-North to IT-Centre-South. The data for the production hubs before removal and the data of IT-Calabria after its addition is aggregated to the bidding zone of IT-South. The bidding zone IT-North, that is considered in the causal model, was not affected.

The aggregated net exports were calculated using the total scheduled exchange considering all neighbours. That is, we add the exports to all neighbouring bidding zones and subtract the imports to all neighbouring bidding zones. We note that we use the total scheduled exchanges instead of the day-ahead scheduled commercial exchange for data quality reasons. In particular, the share of missing data is higher for day-ahead values. In addition Spain has the non-SDAC neighbours Morocco and Andorra. Data of these exchanges can be found using the API of Red Électrica [11].

The residual load is calculated by subtracting day-ahead solar and wind generation from the day-ahead load. ROR generation is hereby neglected to avoid mixing day-ahead and actual generation data.

Since there have been some inconsistencies in ENTSO-E data of unavailable production and generation since the update of the website in the end of 2025, we use alternative sources. In case of France, data on unavailability of nuclear power plants was taken from RTE [12]. Here, we only consider unavailability due to planned maintenance and outages longer than 24 hours, since shorter outages are assumed to not affect the day-ahead price. The nuclear availability is obtained by subtracting the unavailable nuclear capacity from the total installed nuclear capacity. Data on installed nuclear capacity of France is retrieved from ENTSO-E manually with permanent links for reproducability [13]. In case of Spain ESIOS of Red Électrica [14] provides data on accumulated nuclear availability directly, thus not allowing for differentiation between planned and unplanned maintenance. Data of filling rate of Hydro Storage plants and reservoirs is downloaded manually from ENTSO-E manually with permanent links for reproducability [15, 16].The data is available in weekly resolution and is padded to hourly resolution.

Weather data including surface solar radiation downwards, wind speed at 100 m and air

temperature at 2 m is provided by ERA5 [17, 18], mean values are calculated by averaging over larger areas including France and Spain respectively as shown in Supplementary Fig. **11**. The considered area for France is bounded by latitudes 41°-52° N and longitudes 6° W-10° E , and latitudes 36°-44° N and longitudes 10° W-5° E in case of Spain.

The API Hub'Eau [19] provides hourly river temperature data and daily river flow rate data of France. We consider rivers with the highest mean flow rates and those near nuclear power plants: Rhine, Meuse, Rhône, Loire, Garonne, Seine, Dordogne, Vienne and Moselle. For each river, we averaged the data from different measuring stations. Then, we averaged both variables over all rivers. In case of Spain, we used daily river temperature and flow data of the river Ebro as a proxy. The data is provided by SAIH Ebro [20].

All time series used in the analysis are denoted in coordinated universal time (UTC) and have an hourly resolution. Some time series, as for example the gas price, are available only with daily resolution. In this case we padded the daily value for all hours of the day.

SUPPLEMENTARY TABLES

Period	R^2	MAE	RMSE	LMC Violations
**Target: Price day-ahead FR (EUR)**				
2018-01-01 – 2023-12-31	0.92	20.32	30.98	219/352
**Target: Net exports FR (MW)**				
2018-01-01 – 2023-12-31	0.83	1668.69	2133.83	222/352
**Target: Price day-ahead ES (EUR)**				
2018-01-01 – 2023-12-31	0.91	13.63	18.46	171/298

Supplementary Table **1**. Model Performance of SCM's for both electricity market price and net exports as target.

Period	R^2	MAE	RMSE	Mean Label
**Target: Price day-ahead FR (EUR)**				
2018-01-01 – 2023-12-31	0.96	10.71	22.95	96.21
**Target: Net exports FR (MW)**				
2018-01-01 – 2023-12-31	0.92	1236.13	1579.03	4843.20
**Target: Price day-ahead ES (EUR)**				
2018-01-01 – 2023-12-31	0.95	8.03	14.13	79.01

Supplementary Table **2**. GBT performance metrics for both electricity market price and net exports as target.

SUPPLEMENTARY FIGURES

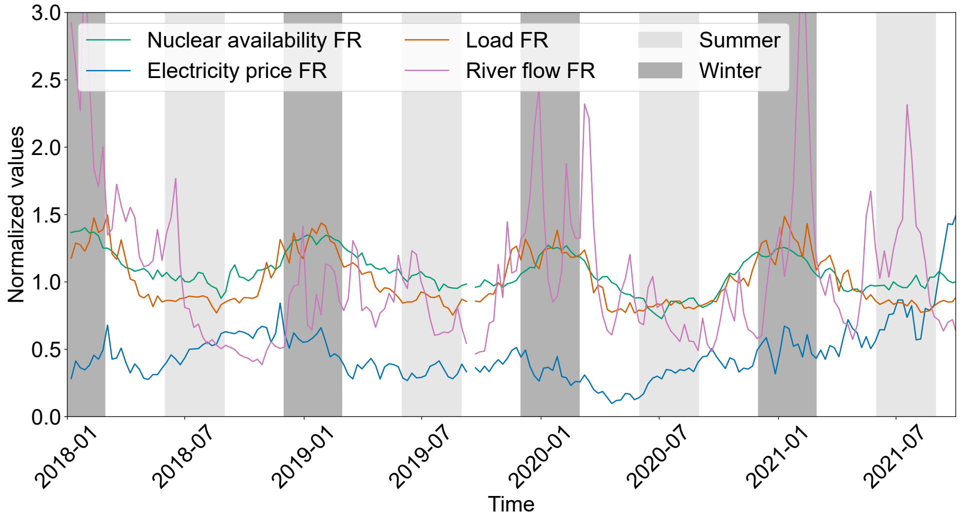

Supplementary Figure 1. **Seasonality in the French electricity system.** We plot the weekly averages of the nuclear availability, river flow, load day-ahead, and the day-ahead electricity market price before the start of the energy crisis normalised by their respective overall means. Winter and Summer are indicated with grey areas. River flow shows strong seasonal patterns. Nuclear availability is highest in the winter, which can be explained by revisions being scheduled in summer when load is lowest. Electricity market prices are higher in case of higher load, causing positive correlation between nuclear availability and electricity market prices.

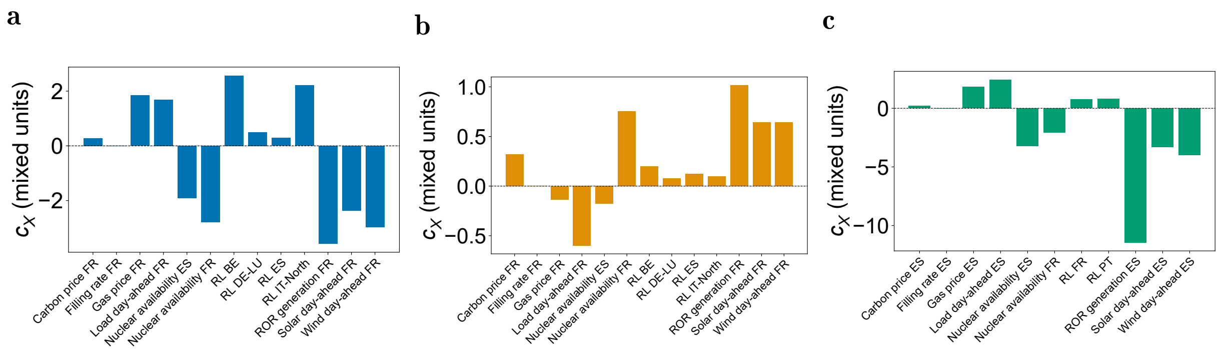

Supplementary Figure 2. **Coefficients of SCM.** Coefficients of SCM with electricity market price of France **a**, net exports of France **b** and electricity market price of Spain **c** as target.

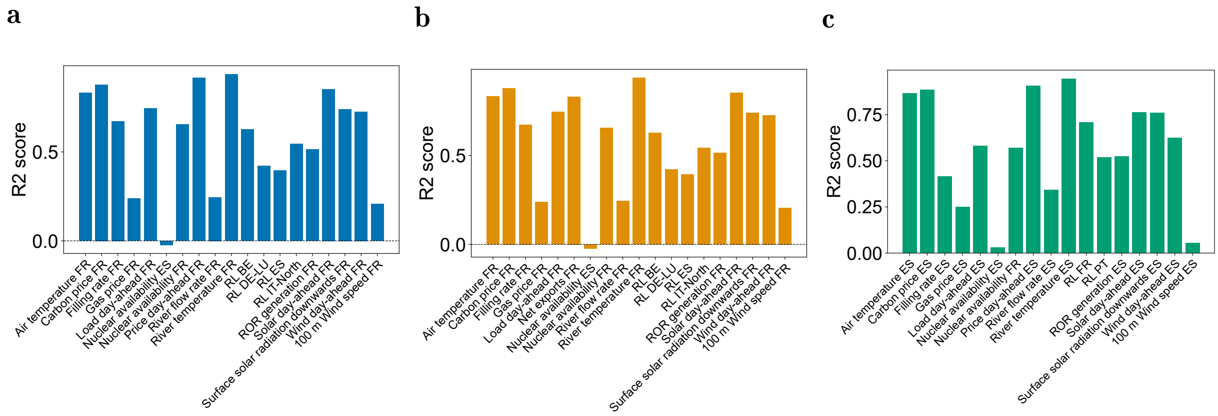

Supplementary Figure 3. **R^2 scores of non-root nodes of SCMs.** R^2 scores of SCM with electricity market price of France **a**, net exports of France **b** and electricity market price of Spain **c** as target. The R^2 score of 0.92 of the French electricity market price and 0.83 of the french net exports and 0.91 of the Spanish electricity market price indicate the suitability of the causal graphs as well as the choice of a linear models.

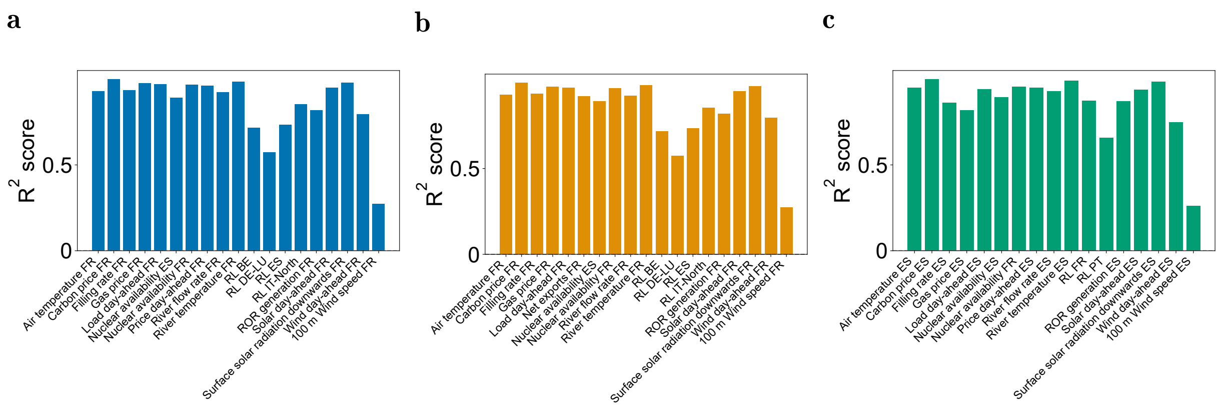

Supplementary Figure 4. **R^2 scores for the GBT models of non-root nodes.** R^2 scores of GBTs with electricity market price of France **a**, net exports of France **b** and electricity market price of Spain **c** as target. The high R^2 scores of the non-root nodes demonstrate the successful explanation by the non-linear GBT models.

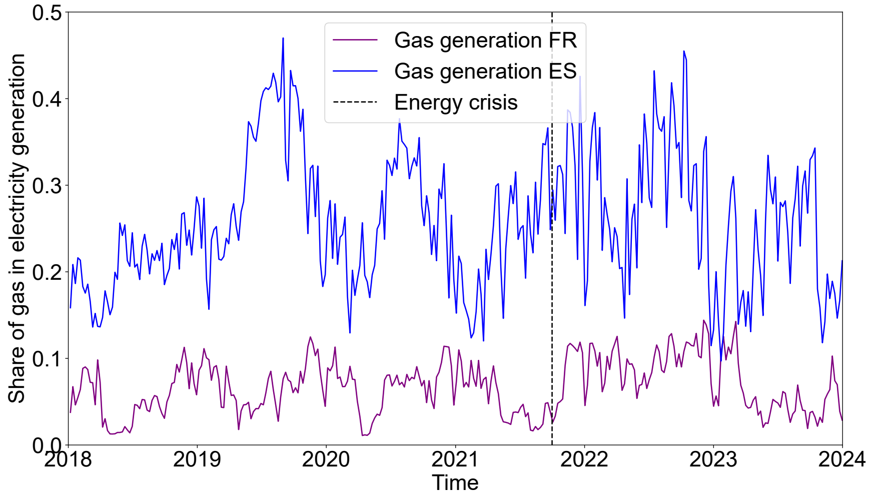

Supplementary Figure 5. **Share of natural gas in french electricity generation.** We plot the weekly average share of gas generation of the total french and Spanish electricity generation. The beginning of the energy crisis defined as 1st October 2021 is marked with a dotted line. The share of gas generation increases after the start of the energy crisis in the year 2022.

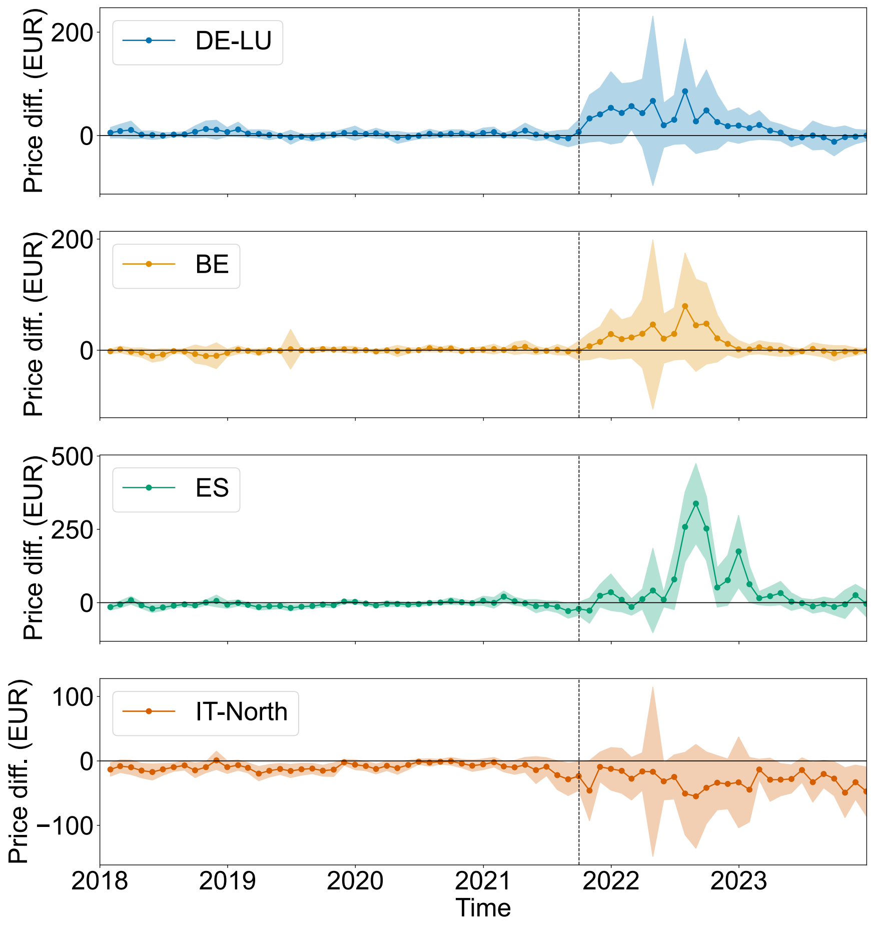

Supplementary Figure 6. Differences of French electricity market prices with respect to the neighbouring bidding zones. We provide a monthly aggregated statistics, where the circles give the mean and the shaded area in indicates the standard deviation. The solid line is drawn to guide the eye. The beginning of the energy crisis defined as 1st October 2021 is marked with a dotted line.

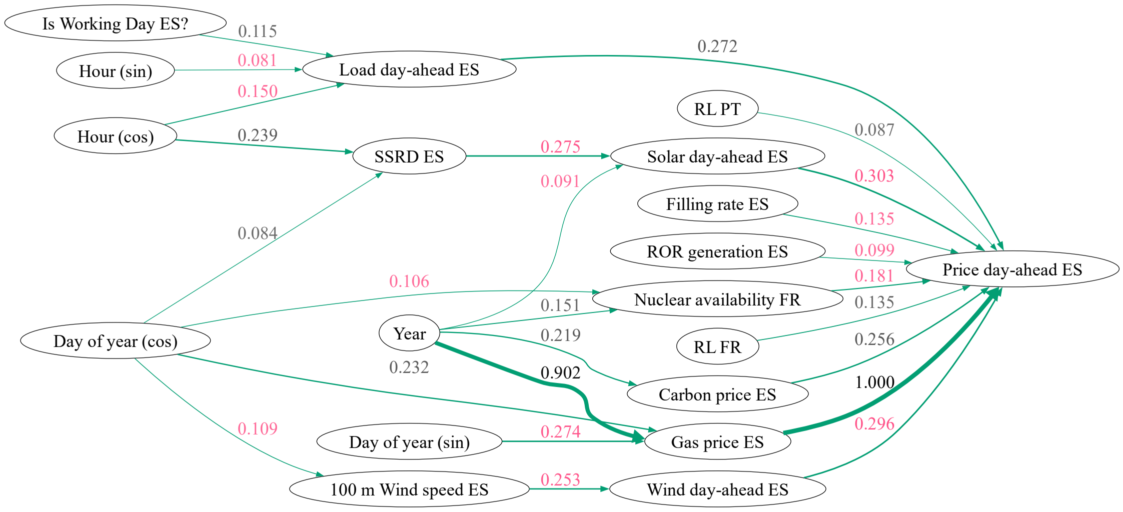

Supplementary Figure 7. **Mean absolute Shapley Flow values explain the non-linear GBT model for the electricity market prices in Spain.** Only the 25 most important edges are shown. The edge attributions quantify how a given feature directly or indirectly influences the target prediction and is given in EUR/MWh. The color indicates a positive (grey) or negative (red) correlation coefficient between Shapley Flow values on the respective edge and the feature value of the starting node.

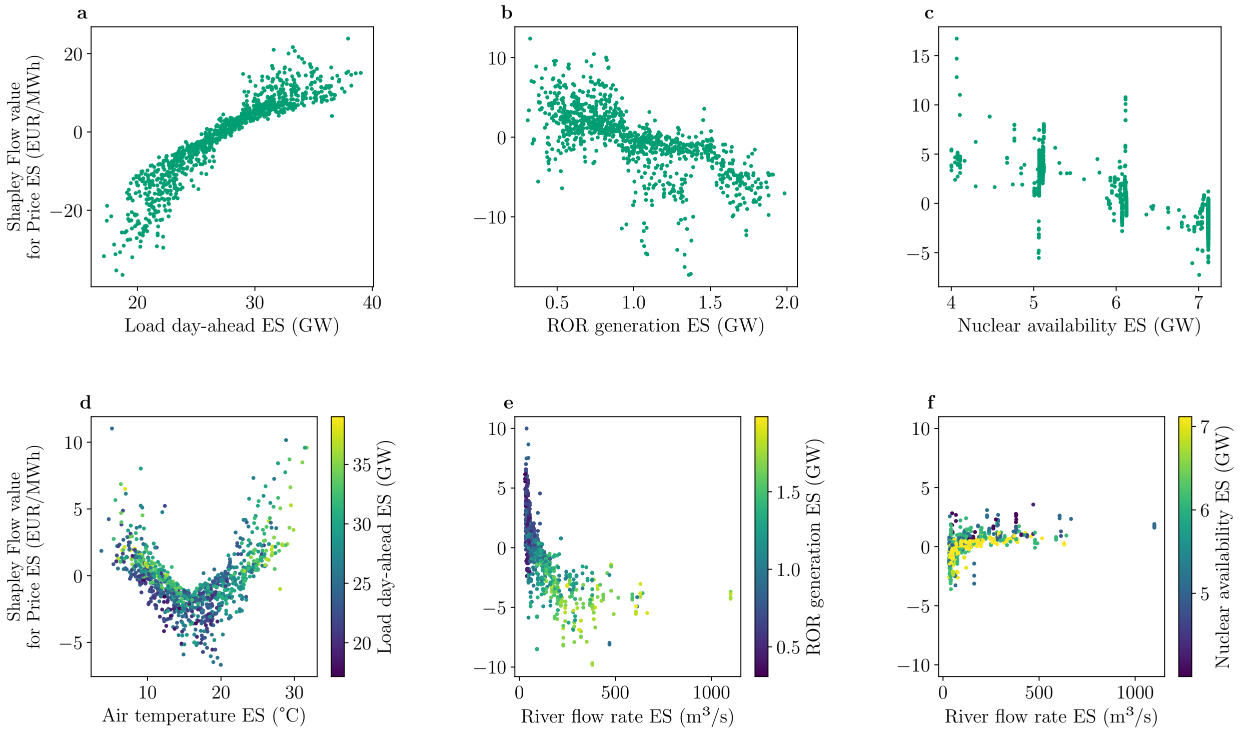

Supplementary Figure 8. **Shapley Flow analysis reveals direct linear and indirect non-linear dependencies. a-c,** Shapley Flow dependencies plots for direct effects in the model for the Spanish electricity market price. **d-f,** Shapley Flow dependencies plots for indirect effects on the electricity market price. Air temperature via Load, River flow rate via ROR generation and River flow rate via Nuclear availability. The value of the intermediate variable is colour coded.

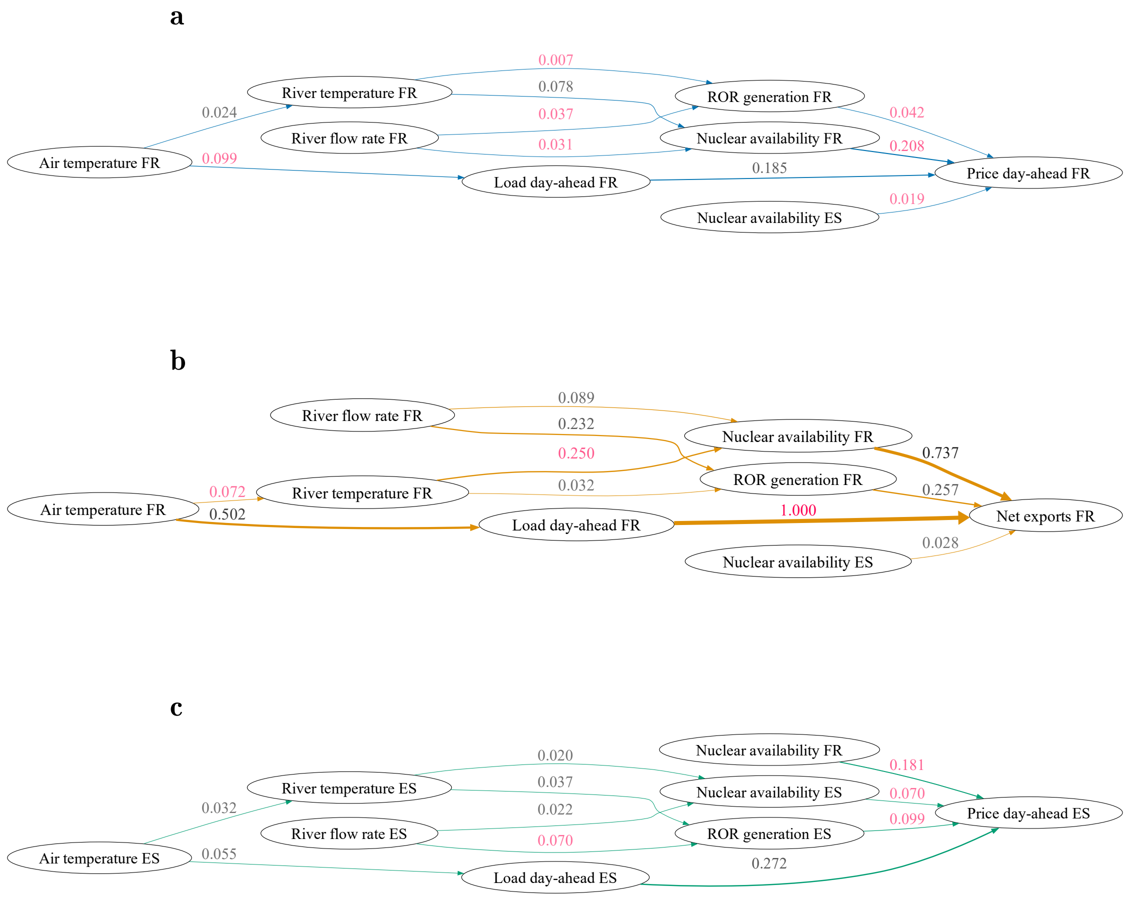

Supplementary Figure 9. **Mean absolute Shapley Flow values for a sub-graph of the causal graph.** **a,** Results for the electricity market price in France. **b,** Results for the net exports of France. **c,** Results for the electricity market price in Spain. We focus on the indirect effects of air temperature, river temperature and river flow rate on the respective target.

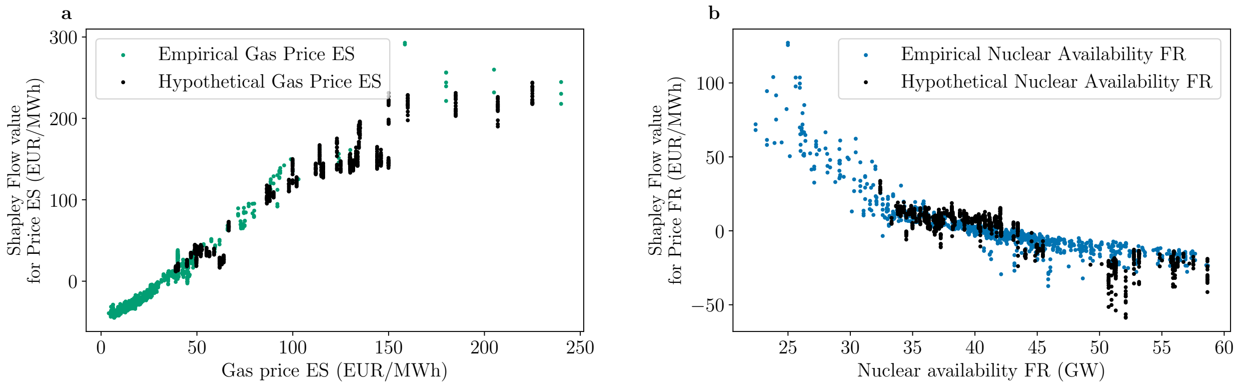

Supplementary Figure 10. **Shapley Flow values for the What-if scenarios. a,** Spanish electricity market price with the hypothetical scenario without a gas price cap. **b,** French electricity market price with the hypothetical scenario of 10 GW additional nuclear availability. We compare Shapley Flow values for the model with original empirical input data (green/blue dots) and hypothetical input data (black). We note that in both what-if scenarios, the value ranges of the Shapley flow values of the hypothetical samples correspond to the ranges of the empirical samples.

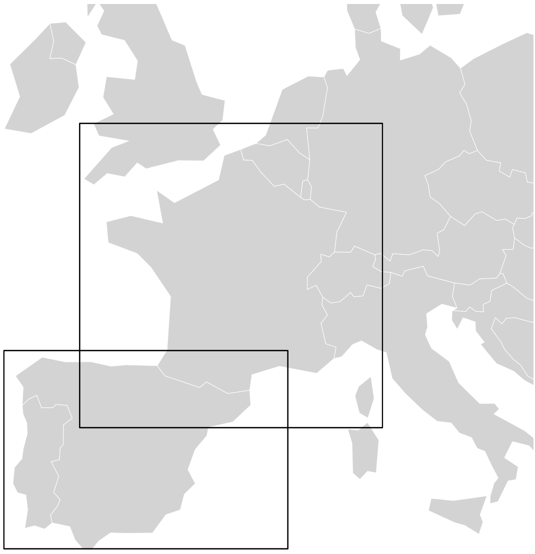

Supplementary Figure 11. **Definition of the weather data.** Weather data is gathered from ERA 5. The areas of France and Spain are hereby approximated with the shown rectangles. Country borders are based on Natural Earth shapefiles [21].

SUPPLEMENTARY REFERENCES

* These authors contributed equally.

† benjamin.schaefer@kit.edu

‡ d.witthaut@fz-juelich.de

[1] Heinen, S., Mancarella, P., O'dwyer, C. & O'malley, M. Heat electrification: The latest research in europe. *IEEE Power and Energy Magazine* **16**, 69–78 (2018).

[2] Sharma, A. & Kiciman, E. Dowhy: An end-to-end library for causal inference. *arXiv preprint arXiv:2011.04216* (2020).

[3] Blöbaum, P., Götz, P., Budhathoki, K., Mastakouri, A. A. & Janzing, D. Dowhy-gcm: An extension of dowhy for causal inference in graphical causal models. *Journal of Machine Learning Research* **25**, 1–7 (2024).

[4] Eulig, E., Mastakouri, A. A., Blöbaum, P., Hardt, M. & Janzing, D. Toward falsifying causal graphs using a permutation-based test. In *Proceedings of the 39th AAAI Conference on Artificial Intelligence*, 26778–26786 (2025).

[5] Pearl, J., Glymour, M. & Jewell, N. P. *Causal inference in statistics: A primer* (John Wiley & Sons, 2016).

[6] Draper, N. & Smith, H. *Applied Regression Analysis* (Wiley, New York, NY, 1998), 3rd edn. Includes disk.

[7] Réseau de Transport d'Électricité. French Annual Electricity Review 2022. `https://analysesetdonnees.rte-france.com/en/electricity-review-keyfindings`. [Accessed 14 Februrary 2025].

[8] Banque de France. Energy balance in 2022: the crisis in nuclear power generation came at the worst possible time. `https://www.banque-france.fr/en/publications-and-statistics/publications/energy-balance-2022-crisis-nuclear-power-generation-came-worst-possible-time` (2023). [Accessed 07 Februrary 2025].

[9] Trebbien, J., Tausendfreund, A., Rydin Gorjão, L. & Witthaut, D. Patterns and correlations in european electricity prices. *Chaos: An Interdisciplinary Journal of Nonlinear Science* **34** (2024).

[10] Trasmissione Elettricità Rete Nazionale. Le nuove zone del mercato elettrico: quello che c'è da sapere. `https://lightbox.terna.it/en/insight/new-electricity-market-zones`. [Accessed 24 January 2024].

[11] Red Eléctrica de España. REE Open Data API. `https://www.ree.es/en/apidatos`. Accessed 29 January 2026.

[12] Réseau de Transport d'Électricité (RTE). Production Unavailability data from RTE Inside Information Platform. `https://iip.cloud-rte-france.com/production-unavailability`. Accessed 22 December 2025.

[13] ENTSO-E. Installed capacity per production type france. https://transparency.entsoe.eu/ (2025). Data retrieved via ENTSO-E Transparency Platform permalinks for multiple timeframes: 2018–2020: https://transparency.entsoe.eu/generation/installed/perType?permalink=691f25daabdb9a19ccd6eb73; 2021–2023: https://transparency.entsoe.eu/generation/installed/perType?permalink=691f26583936c4051e5916e2. Accessed 20 November 2025.

[14] Red Eléctrica de España (REE). ESIOS Analysis data from https://www.esios.ree.es/en/analysis. https://www.esios.ree.es/en/analysis. Accessed 09 January 2026.

[15] ENTSO-E. Water reservoirs and hydro storage plants france. https://transparency.entsoe.eu/ (2025). Data retrieved via ENTSO-E Transparency Platform permalinks for multiple timeframes: 2018–2020: https://transparency.entsoe.eu/generation/hydroReserve?permalink=691f2828dc8b9d780681e46b; 2021–2023: https://transparency.entsoe.eu/generation/hydroReserve?permalink=691f28733936c4051e5916e6. Accessed 20 November 2025.

[16] ENTSO-E. Water reservoirs and hydro storage plants spain. https://transparency.entsoe.eu/ (2025). Data retrieved via ENTSO-E Transparency Platform permalinks for multiple timeframes: 2018–2020: https://transparency.entsoe.eu/generation/hydroReserve?permalink=691f28b43936c4051e5916e9; 2021–2023: https://transparency.entsoe.eu/generation/hydroReserve?permalink=691f290cdc8b9d780681e5f6. Accessed 20 November 2025.

[17] Copernicus Climate Change Service, Climate Data Store. ERA5 hourly data on single levels from 1940 to present (2023). Accessed 11–12 November 2025.

[18] Hersbach, H. *et al.* ERA5 hourly data on single levels from 1940 to present (2023). Accessed 11–12 November 2025.

[19] Office français de la biodiversité. Eau france: Hub'eau. https://hubeau.eaufrance.fr/. [Accessed 29 January 2026].

[20] Confederación Hidrográfica del Ebro (CHE). SAIH Ebro historical hydrological data. https://www.saihebro.com/datos/historicos. Accessed 15 October 2025.

[21] Natural Earth. Natural earth. free vector and raster map data (2026). URL https://www.naturalearthdata.com/.
