# Supplementary material for: Understanding the European energy crisis through structural causal models
Source: Nat Commun. 2026 Jul 17;17:6539. doi: 10.1038/s41467-026-75433-7 (PMC13379379; doi:10.1038/s41467-026-75433-7)
Supplement: Supplementary file 2 — Transparent Peer Review file [file 41467_2026_75433_MOESM2_ESM.pdf]

nature portfolio

Peer Review File

Understanding the European energy crisis through structural causal models

Corresponding Author: Professor Dirk Witthaut

This file contains all reviewer reports in order by version, followed by all author rebuttals in order by version.

Version 0:

Reviewer comments:

Reviewer #1

(Remarks to the Author)
I appreciate the opportunity to review the manuscript, “Understanding the European energy crisis through structural causal models.” The study is a strong and timely contribution, applying causal inference and interpretable machine learning to a pressing policy and scientific problem.

The use of structural causal models alongside gradient boosted trees with Shapley Flow provides insights into electricity market dynamics during the European energy crisis. This dual approach (transparent linear models + flexible non-linear machine learning) is very valuable.

The analysis convincingly addresses Simpson’s paradox in the correlation between nuclear availability and electricity prices, restoring consistency with economic theory.

The paper identifies the interplay between soaring natural gas prices and low nuclear availability as the primary drivers of French electricity price spikes, supported by strong quantitative evidence.

The study relies on transparent data sources such as ENTSO-E and code availability enhances reproducibility and transparency which are important in open science practices.

That said, I encourage you to consider the following revisions before publication:

1) The SCM evaluation reports a relatively high number of LMC violations (172/236). Although the models outperform random permutations, this level of violation suggests fragility in the assumed causal structure. More discussion of alternative desired graphs and robustness checks would strengthen credibility.

2) While the GBT analysis partially compensates, the reliance on linear SCMs risks oversimplifying inherently non-linear and feedback-driven relationships for example between outages and price formation. The manuscript should more clearly acknowledge and discuss these limitations.

3) Key factors such as government interventions (subsidies, caps, demand reduction measures) and unplanned nuclear outages are not explicitly modeled, though they clearly affected prices. Sensitivity analysis or at least a more explicit discussion is needed.

4) The relatively low explanatory power for certain intermediate nodes (nuclear availability, R2 = 0.44) raises concerns about omitted drivers. This should be discussed more openly in the text.

5) The analysis is tightly focused on France. While appropriate, the manuscript would benefit from explicit discussion of how findings might (or might not) generalize to other European contexts.

The manuscript offers a valuable and original contribution to the empirical study of energy markets during crisis conditions. Its methodological combination of SCMs and interpretable ML is timely and promising. However, the current version

overstates the conclusiveness of its causal claims and requires more robust treatment of alternative explanations and model limitations.

(Remarks on code availability)

Reviewer #2

(Remarks to the Author)
The authors present a two-pronged approach to analyze the drivers of French wholesale electricity prices and net exports. They employ a linear structural causal model to establish a causal graph and then use a non-linear, tree-based machine learning model combined with Shapley Flow values to quantify these relationships. The implementation of the proposed methods appears to be sound and correctly executed based on the descriptions provided. However, I see some weaknesses in the discussion of the model's practical utility. While the models confirm known phenomena from the 2022 energy crisis, the paper does not articulate what novel insights can be gained from this sophisticated analysis.

One aspect discussed intensely is Simpson’s paradox, which was found to apply in the relationship between nuclear availability and wholesale prices and net exports, respectively. Are the values of 0.4461 and -0.0015 the slopes of the regression functions or the Pearson’s correlation coefficient values? The caption says slopes, but the text argues about correlations. Adding a goodness-of-fit metric like R^2 values would be desirable for an evaluation. Furthermore, a crucial point not considered is the potential for reverse causality between nuclear availability and electricity prices. Since planned maintenance is included in outages and is often scheduled during periods of anticipated low prices, low prices can be said to "cause" these outages. This is a critical distinction, as the dynamics of planned outages differ significantly from the unplanned outages of the 2022 crisis. Finally, the statement that an observed positive correlation is in "clear violation of economic laws" is a significant overstatement. Correlation can neither confirm nor falsify a causal law.

The manuscript's explanation of the causal graph is confusing, particularly regarding the indirect path between nuclear availability and price. The authors state that "this indirect path is not causal; in the graph we have to pass an arrow against its causal direction," which is an unclear statement that requires clarification. The authors should explain what "its" refers to and illustrate this concept within Figure 2b. Additionally, the claim that "the indirect path essentially determines the correlations" seems to obscure the fact that the load (which differs across the time of the year) has a much stronger impact on prices than the nuclear availability.

A shortcoming of the paper is its failure to demonstrate the value of the model beyond confirming the obvious. The conclusion that high gas prices and low nuclear availability led to high electricity prices in France is a widely understood fact of the 2022 energy crisis. The central question is what new insights this complex model provides. To strengthen the manuscript, the authors should address the practical applications and interpretive power of their results. For instance, they should explain how the coefficients in Figure 4 or the Shapley flows in Figure 6 could be used for what-if scenario analysis, whether this framework could discover non-obvious relationships if applied to another country like Norway (which the authors identify as a country that also experienced high price increases during the crisis), and how one should interpret non-trivial direct causal flows, such as the one from air temperature to net exports.

Several minor points also warrant attention. In the introduction, the authors argue that “the design of the future energy system remains highly controversial, particularly with regard to the role of nuclear power plants […]. Empirical research on electricity prices and their drivers is needed to enable informed decisions.” I don’t see how the one relates to the other. Of course, knowledge on the drivers of electricity prices is always helpful, but how does this knowledge contribute to energy system transformation research? The latter typically doesn’t focus on wholesale electricity prices.

The argument that data-driven approaches become necessary “to handle the enormous amount of available information” is questionable. Doesn't the necessity of data processing come from the demand for system understanding rather than from available data itself? Data-driven approaches create INFORMATION from DATA; the terms should be distinguished. The authors then argue that data analysis “should not be limited to black-box predictions or correlation analysis but needs to inform interventions”. The one doesn’t contradict the other, and all methods must be chosen in relation to the research objective.

Due to the limited discussion of the usefulness of the model, I would suggest rejecting the paper.

(Remarks on code availability)

Version 1:

Reviewer comments:

Reviewer #2

(Remarks to the Author)
In the revised version, many aspects have been improved and some unclear parts are now better explained. The inclusion of the what-if-analysis increases the value of the paper. However, when discussing the results, the authors mainly describe

relationships that are all well known among electricity market modelers qualitatively, so the model would deliver the additonal value of quantifying the studied impacts. But for trusting the quantification, some evaluation of the model quality, using the usual error metrics would be needed, not only measures like R^2. Therefore, I remain sceptical about the value of the described model in comparison to fundamental market models on the one hand and machine learning models on the other hand. But the concept itself is well presented and is illustrated in high quality.

(Remarks on code availability)

Open Access This Peer Review File is licensed under a Creative Commons Attribution 4.0 International License, which permits use, sharing, adaptation, distribution and reproduction in any medium or format, as long as you give appropriate credit to the original author(s) and the source, provide a link to the Creative Commons license, and indicate if changes were made.
In cases where reviewers are anonymous, credit should be given to 'Anonymous Referee' and the source.
The images or other third party material in this Peer Review File are included in the article’s Creative Commons license, unless indicated otherwise in a credit line to the material. If material is not included in the article’s Creative Commons license and your intended use is not permitted by statutory regulation or exceeds the permitted use, you will need to obtain permission directly from the copyright holder.
To view a copy of this license, visit https://creativecommons.org/licenses/by/4.0/

REPLIES TO THE REVIEWER COMMENTS.

First, we thank the reviewers for their positive and constructive feedback. We apologise for the delay in the revision of this manuscript. Our main data sources, the HUB'EAU [1] and the ENTSO-E transparency platform [2] underwent significant changes during the time between the first submission and the first round of review. This required us to comprehensively revise our software in order to ensure continued access to the raw data and the reproducibility of our results.

In the following, we respond to each comment in detail. Reviewer comments are reproduced in italics, our replies are given in blue (Our reply: sample reply), and changes to the manuscript are indicated in red (Changes to the manuscript: sample change).

We have included additional figures in the main manuscript and several additional figures in the supplement, such that the numbering has changed. References in our replies refer to the new numbering in the revised version.

REVIEWER 1

I appreciate the opportunity to review the manuscript, "Understanding the European energy crisis through structural causal models." The study is a strong and timely contribution, applying causal inference and interpretable machine learning to a pressing policy and scientific problem. The use of structural causal models alongside gradient boosted trees with Shapley Flow provides insights into electricity market dynamics during the European energy crisis. This dual approach (transparent linear models + flexible non-linear machine learning) is very valuable. The analysis convincingly addresses Simpson's paradox in the correlation between nuclear availability and electricity prices, restoring consistency with economic theory. The paper identifies the interplay between soaring natural gas prices and low nuclear availability as the primary drivers of French electricity price spikes, supported by strong quantitative evidence. The study relies on transparent data sources such as ENTSO-E and code availability enhances reproducibility and transparency which are important in open science practices.That said, I encourage you to consider the following revisions before publication:

Our reply: We thank the reviewer for their interest in our work and the constructive comments on our manuscript.

1) The SCM evaluation reports a relatively high number of LMC violations (172/236). Although the models outperform random permutations, this level of violation suggests fragility in the assumed causal structure. More discussion of alternative desired graphs and robustness checks would strengthen credibility.

Our reply: We thank the reviewer for this thoughtful comment and for pointing out that the relatively high number of LMC violations should be discussed.

Following the reviewer's comment, we have modified our causal models by including additional features (like year, further meteorological features, filling rate of hydro storage and nuclear availability of a neighbouring country), thus providing a more detailed model. This modification led to an improvement in the relative number of LMC violations and reached $227/352 \approx 0.64$ for the model of French electricity prices and $228/352 \approx 0.65$ for French net exports, compared to $172/236 \approx 0.73$ before.

Importantly, these structural refinements do not materially alter the main conclusions regarding electricity prices and net exports. The qualitative ranking and quantitative contributions of the key drivers remain stable across model specifications.

We note that even though LMC violations appear high, fewer are observed than in all 50 randomly permuted graphs tested during model validation. This is indicated by a p-value of 0.

Changes to the manuscript:

- We have extended the causal model to include additional variables as shown in Figure 3 to improve model performance.
- Accordingly, we have updated the discussion of the model performance in the supplementary information.

2) While the GBT analysis partially compensates, the reliance on linear SCMs risks oversimplifying inherently non-linear and feedback-driven relationships for example between outages and price formation. The manuscript should more clearly acknowledge and discuss these limitations.

Our reply: We agree that the linear SCMs cannot capture non-linear effects in the data. We have thus decided to base our analysis of indirect causal effects on the GBT model, as nonlinearity clearly plays an important role here. Furthermore, we have revised the manuscript to clarify this aspect as suggested by the reviewer.

We remark that the GBT models confirm that the final targets price and net exports are well captured by linear models. This is confirmed by a comparison of R^2 scores of both the linear and the GBT models provided in the Supplementary Information. Scores are very similar for the final targets, but differ considerably for some intermediate nodes.

Changes to the manuscript:

- We have strongly revised the analysis of indirect effect of river flow and temperature. The revised version relies on the GBT model and Shapley Flow values to account for nonlinear dependencies. Hence, we have deleted the old figure on indirect effects and expanded figure 6 instead. Furthermore we have added the new Supplementary Figure 9.
- We conclude the introduction of the SCM by the sentence: "The linear formulation enables a transparent and interpretable quantification of direct causal effects. To assess potential non-linearities and indirect effects, we complement this analysis with a Shapley Flow approach below. "
- We have added the following paragraph discussing the strengths and weaknesses of the linear SCM: "We fit the proposed linear SCM to French and Spanish data covering the period 2018–2023. For electricity market prices, the models achieve R^2 scores of 0.92 and 0.91, respectively (Supplementary Tab. 1), indicating strong explanatory power. In contrast, the R^2 values for intermediate variables vary substantially (Supplementary Fig. 3). Lower values suggest either limitations of the linear approximation or the presence of relevant exogenous drivers not included in the model. For example, wind power generation is largely determined by synoptic meteorological conditions that are not explicitly modelled."

3) Key factors such as government interventions (subsidies, caps, demand reduction measures) and unplanned nuclear outages are not explicitly modeled, though they clearly affected prices. Sensitivity analysis or at least a more explicit discussion is needed.

Our reply: We thank the reviewer for this comment. Indeed, causal models are essential to study the effect of interventions and we are happy to extend our analysis into this direction.

During the revision, we carried out extensive additional analysis leading to a new section on "What-If scenarios". There, we use our GBT model to analyse two hypothetical situations. First, we examine the Iberian exception, i.e. the impact of a gas price cap on the Spanish electricity prices. To this end we compare empirical and predicted prices with and without the government intervention. Notably, our results agree very well with results from mechanistic market models reported in the literature. Second, we study the hypothetical situation of "normal" nuclear availability in France in 2022.

Changes to the manuscript:

- We have added a detailed analysis of a government intervention, a gas price cap commonly referred to as "Iberian exception".
- We have added a hypothetical scenario of "normal" nuclear availability in France in 2022.
- We have added a new figure 7 and a new section summarizing our results on the What-If scenarios.
- We have added further references on the Iberian exception.

4) The relatively low explanatory power for certain intermediate nodes (nuclear availability, R2 = 0.44) raises concerns about omitted drivers. This should be discussed more openly in the text.

Our reply: We fully agree that the explanatory power of certain intermediate variables has been mediocre. Following the reviewer's comment, we have thus extended the causal model to include additional variables as described above and we have expanded GBT model that accounts for non-linear dependencies. These measures have led to an improvement of R^2 scores as reported in Supplementary Fig. 3 and Supplementary Fig. 4.

For certain intermediate nodes, the performance remains limited. The most striking example is wind power generation and the residual load in the DE-LU bidding zone that depends heavily on wind power generation. This result is fully expected as wind power is crucially determined by synoptic meteorological variables. We decided to not include these variables in order to maintain focus.

The R^2 score of nuclear availability of France has improved to $R^2 = 0.66$ in the extended linear SCM. Hence, roughly one third of the variability remains exogenous to the model. It has been reported in the media that delayed maintenance and corrosion problems affected the French nuclear fleet in 2022. These factors are not represented explicitly in the manuscript because reliable quantitative information is sparse.

From the perspective of structural causal modelling, these unknown effects are attributed to exogenous variables. A low R^2 score thus indicates a strong role of exogenous effects. We have modified the manuscript to communicate this aspect more clearly.

Changes to the manuscript:

- We revised the model itself as shown in Figure 3 of the main text to improve model performance.
- We use the GBT model for the analysis of indirect effects, which shows a better performance for certain intermediate nodes.
- We report the R^2 scores of both models in Supplementary Fig. 2 and Supplementary Fig. 3. We have added a paragraph discussing the performance of the model in more detail: "We fit the proposed linear SCM to French and Spanish data covering the period 2018–2023. For electricity market prices, the models achieve R^2 scores of 0.92 and 0.91, respectively (Supplementary Tab. 1), indicating strong explanatory power. In contrast, the R^2 values for intermediate variables vary substantially (Supplementary Fig. 3). Lower values suggest either limitations of the linear approximation or the presence of relevant exogenous drivers not included in the model. For example, wind power generation is largely determined by synoptic meteorological conditions that are not explicitly modelled."

5) The analysis is tightly focused on France. While appropriate, the manuscript would benefit from explicit discussion of how findings might (or might not) generalize to other European contexts.

Our reply: We thank the reviewer for this very helpful comment and we are happy to extend our manuscript accordingly. As part of our extensive revision, we added a causal analysis of electricity prices in Spain in addition to France. This extension allows us to analyze two policy relevant aspects: (i) the "spillover" of nuclear problems from France to Spain due to market coupling and (ii) the effect of the Spanish gas price cap on electricity prices as discussed above.

Furthermore, we have revised the discussion section accordingly. We discuss the generalization explicitly in the outlook with data availability as a limiting factor.

Changes to the manuscript:

- We added a causal analysis of electricity prices in Spain in addition to France.
- We have updated all figures accordingly. Spanish data has been added to Figure 1. Results of the linear SCM for Spain have been included in Figure 4. Results of the analysis of the Iberian exception are presented in Figure 7. Shapley flow values for Spanish electricity prices are reported in Supplementary Fig. 7.
- We have added a new Section "Cross-border effects" to the main manuscript focusing on electricity market price in the Spanish market. A key result is how changes in the French bidding zone affected Spanish prices.
- We have extended the main text to discuss the Iberian exception and compare our result to the literature in the new Section "What-If Scenarios"
- We have added references on the Iberian exception.

REVIEWER 2

The authors present a two-pronged approach to analyze the drivers of French wholesale electricity prices and net exports. They employ a linear structural causal model to establish a causal graph and then use a non-linear, tree-based machine learning model combined with Shapley Flow values to quantify these relationships. The implementation of the proposed methods appears to be sound and correctly executed based on the descriptions provided. However, I see some weaknesses in the discussion of the model's practical utility. While the models confirm known phenomena from the 2022 energy crisis, the paper does not articulate what novel insights can be gained from this sophisticated analysis.

Our reply: We thank the reviewer for the critical and constructive assessment of our work. In our extensive revision, we have made an effort to clarify which novel insights are accessible with our analysis, including a new section on a "what-if" analysis. We detail these changes below.

One aspect discussed intensely is Simpson's paradox, which was found to apply in the relationship between nuclear availability and wholesale prices and net exports, respectively. Are the values of 0.4461 and -0.0015 the slopes of the regression functions or the Pearson's correlation coefficient values? The caption says slopes, but the text argues about correlations. Adding a goodness-of-fit metric like R^2 values would be desirable for an evaluation.

Our reply: We agree that using both coefficients and slopes is confusing. Given the low R^2 values of the linear fits, Pearson's correlation coefficients are a more appropriate measure. We have decided to keep the linear fits in the plots for ease of interpretation.

Changes to the manuscript:

- We have replaced slopes by Pearson's correlation coefficients in Figure 2 and we have revised the text accordingly.

Furthermore, a crucial point not considered is the potential for reverse causality between nuclear availability and electricity prices. Since planned maintenance is included in outages and is often scheduled during periods of anticipated low prices, low prices can be said to "cause" these outages. This is a critical distinction, as the dynamics of planned outages differ significantly from the unplanned outages of the 2022 crisis.

Our reply: We thank the reviewer for this constructive comment and have modified our presentation of Simpson's paradox accordingly. In particular, we agree that the previous argument based on "time of year" was not entirely clear.

Considering the potential for reverse causality, we concur that planned outages are typically scheduled during periods of expected low prices due to *expected low load*. However, expected prices are not identical to realized day-ahead prices. We have thus revised the presentation replacing the vague "time of year" by the clearer "expected load". In this way, the chain of causation mentioned by the reviewer is in fact included in the stylized causal graph in Fig. 2b.

Changes to the manuscript:

- We modified the stylized causal graph in Fig. 2b explaining the paradoxical behaviour, replacing "time of year" by "expected load".
- We have revised the corresponding paragraph in the main text as discussed below.

Finally, the statement that an observed positive correlation is in "clear violation of economic laws" is a significant overstatement. Correlation can neither confirm nor falsify a causal law.

Our reply: We fully agree and we have revised the manuscript accordingly.

Changes to the manuscript:

- We have replaced "in clear violation of economic laws" by "counterintuitive to economic laws".

The manuscript's explanation of the causal graph is confusing, particularly regarding the indirect path between nuclear availability and price. The authors state that "this indirect path is not causal; in the graph we have to pass an arrow

against its causal direction," which is an unclear statement that requires clarification. The authors should explain what "its" refers to and illustrate this concept within Figure 2b. Additionally, the claim that "the indirect path essentially determines the correlations" seems to obscure the fact that the load (which differs across the time of the year) has a much stronger impact on prices than the nuclear availability.

Our reply: We thank the reviewer for pointing out the shortcoming of our explanation. We have thoroughly revised the respective paragraph to improve clarity.

Changes to the manuscript:

- We have revised the corresponding paragraph as follows: "This situation is illustrated in Fig. 2a,b, where arrows represent hypothesized causal effects. The relationship between nuclear availability and electricity market prices is governed by two paths: (i) a direct causal effect of availability on prices, consistent with the law of supply, whereby higher available capacity tends to reduce prices, and (ii) an indirect path via expected and realized load. Expected load influences planned outages, as maintenance is typically scheduled during periods of forecasted low demand and low prices. At the same time, expected load strongly predicts the realized day-ahead load, which in turn affects prices. Expected load therefore acts as a confounder: periods of high expected load are associated with both higher nuclear availability and higher prices. This induces a positive correlation between availability and price, even though the direct causal effect of additional nuclear supply would reduce prices."

A shortcoming of the paper is its failure to demonstrate the value of the model beyond confirming the obvious. The conclusion that high gas prices and low nuclear availability led to high electricity prices in France is a widely understood fact of the 2022 energy crisis. The central question is what new insights this complex model provides. To strengthen the manuscript, the authors should address the practical applications and interpretive power of their results. For instance, they should explain how the coefficients in Figure 4 or the Shapley flows in Figure 6 could be used for what-if scenario analysis, whether this framework could discover non-obvious relationships if applied to another country like Norway (which the authors identify as a country that also experienced high price increases during the crisis), and how one should interpret non-trivial direct causal flows, such as the one from air temperature to net exports.

Our reply: We thank the reviewer for this comment. During the revision, we carried out extensive additional analysis to obtain additional "non-obvious" insights. Before diving into details, we note that although several mechanisms of the energy crisis were qualitatively known, their relative importance and interaction can only be assessed through quantitative analysis. Our framework provides such an assessment.

Following the reviewer's advice regarding generalizability, we extended our manuscript by applying the framework to a second national market and developing an additional model for Spanish electricity prices. This extension demonstrates that the proposed methodology is transferable beyond a single-country case study. Moreover, it allows us to analyse how shocks in the French electricity system during the energy crisis propagated to Spain, thereby quantifying cross-border transmission effects within the same causal framework. In addition, we have clarified the interpretation of non-trivial causal flows, such as the influence of air temperature on net exports, by explicitly linking them to load dynamics.

Second, we have added a new section on "What-if scenarios" as suggested by the reviewer. We investigate two hypothetical questions: What if Spain had not issued a gas price cap during the energy crisis? What if ten additional nuclear reactors had been available in France in 2022? We conclude that the gas price cap has been effective in reducing electricity market prices and compare our results to the literature. Furthermore, we observe that ten additional nuclear reactors in France would have had a limited effect in most hours, with drastic reductions in electricity prices only during the summer of 2022. Loosely speaking, ten additional reactors would not have been sufficient to replace gas-fired plants or imports completely during most of 2022. Hence, gas-fired plants would have still played a decisive role in price formation.

Changes to the manuscript:

- We have implemented an additional model for electricity market prices in Spain, cf. Fig. 4 and Supplementary Fig. 7.

- We have revised the analysis and discussion of non-trivial (indirect) causal flows from meteorological variables to the target. The revised analysis is based on the non-linear model and Shapley Flows; we have thus extended Fig. 6.

- We have added a new section "cross-border effects" focusing on the effect of the French electricity system on the electricity market price in Spain.
- We have added a new section on "What-If scenarios" and a new Figure 7.
- We have revised the discussion accordingly.

Several minor points also warrant attention. In the introduction, the authors argue that "the design of the future energy system remains highly controversial, particularly with regard to the role of nuclear power plants [...]. Empirical research on electricity prices and their drivers is needed to enable informed decisions." I don't see how the one relates to the other. Of course, knowledge on the drivers of electricity prices is always helpful, but how does this knowledge contribute to energy system transformation research? The latter typically doesn't focus on wholesale electricity prices.

Our reply: We thank the reviewer for this helpful comment. Indeed, our initial argument was not well-connected and we are happy to revise the introduction according to the reviewer's suggestion. We argue that political debates concern the consequences of concrete *interventions*, such as the Spanish gas price cap during the energy crisis. Structural causal models are ideally suited for this task as they show how structural changes propagate through electricity systems.

Changes to the manuscript:

- We have revised the respective text as follows: "In general, debates about the future energy system remain highly controversial, in particular with regard to the role of nuclear power plants [3, 4] and renewable energy sources [5, 6]. At their core, these debates concern the consequences of concrete interventions, such as capacity expansion, plant closures, or market regulation. Assessing such consequences requires empirical evidence on how structural changes propagate through electricity systems and affect market outcomes."

The argument that data-driven approaches become necessary "to handle the enormous amount of available information" is questionable. Doesn't the necessity of data processing come from the demand for system understanding rather than from available data itself? Data-driven approaches create INFORMATION from DATA; the terms should be distinguished. The authors then argue that data analysis "should not be limited to black-box predictions or correlation analysis but needs to inform interventions". The one doesn't contradict the other, and all methods must be chosen in relation to the research objective.

Our reply: We thank the reviewer for pointing out this inconsistency in our initial submission. We have removed the questionable statements from the introduction and we have rewritten the respective paragraph. The key point is that the availability of comprehensive data sets enables a systematic statistical analysis. Causal models identify the effects of interventions and are thus particularly suited for informing the public debate.

Changes to the manuscript:

- We have revised the respective text as follows: "The increasing availability of detailed operational and market data [2] enables systematic analysis of such complex interactions [7, 8]. However, while predictive models and correlation analyses can describe patterns, they do not by themselves identify the effects of interventions. To inform system design and policy choices, causal explanations are required [9]."

FURTHER CHANGES

Changes to the manuscript:

- We have thoroughly revised the data processing code to reflect recent changes to the HUB'EAU [1] and ENTSO-E transparency platform [2]. In this context, we also updated and completed the corresponding data sets.
- We have updated the Methods section and the Supplementary Information accordingly.
- We have removed the former Fig. 8 from the Methods section.

[1] Office français de la biodiversité, “Eau france: Hub’eau,” https://hubeau.eaufrance.fr/, [Accessed 29 January 2026].
[2] European Network of Transmission System Operators for Electricity, “Electricity Market Transparency — entsoe.eu,” https://www.entsoe.eu/data/transparency-platform/, [Accessed 29 January 2026].
[3] J. D. Jenkins, M. Luke, and S. Thernstrom, Joule **2**, 2498 (2018).
[4] L. Haywood, M. Leroutier, and R. Pietzcker, Joule **7**, 1675 (2023).
[5] M. Pahle, O. Tietjen, S. Osorio, F. Egli, B. Steffen, T. S. Schmidt, and O. Edenhofer, Nature Energy **7**, 290 (2022).
[6] T. Brown and L. Reichenberg, Energy Economics **100**, 105354 (2021).
[7] M. Khodayar, G. Liu, J. Wang, and M. E. Khodayar, CSEE Journal of Power and Energy Systems **7**, 209 (2020).
[8] W. Strielkowski, A. Vlasov, K. Selivanov, K. Muraviev, and V. Shakhnov, Energies **16**, 4025 (2023).
[9] B. Schölkopf, F. Locatello, S. Bauer, N. R. Ke, N. Kalchbrenner, A. Goyal, and Y. Bengio, Proceedings of the IEEE **109**, 612 (2021).

REPLIES TO THE REVIEWER COMMENTS.

First, we thank the reviewers for their positive and constructive feedback.

In the following, we respond to each comment in detail. Reviewer comments are reproduced in italics, our replies are given in blue (Our reply: sample reply), and changes to the manuscript are indicated in red (Changes to the manuscript: sample change).

REVIEWER 2

In the revised version, many aspects have been improved and some unclear parts are now better explained. The inclusion of the what-if-analysis increases the value of the paper. However, when discussing the results, the authors mainly describe relationships that are all well known among electricity market modelers qualitatively, so the model would deliver the additonal value of quantifying the studied impacts. But for trusting the quantification, some evaluation of the model quality, using the usual error metrics would be needed, not only measures like R^2. Therefore, I remain sceptical about the value of the described model in comparison to fundamental market models on the one hand and machine learning models on the other hand. But the concept itself is well presented and is illustrated in high quality.

Our reply: We thank the reviewer for the constructive comments and for pointing out that additional error metrics would strengthen confidence in the quantitative results. Following this suggestion, we have extended the model evaluation in the Supplementary Information by reporting the root mean squared error (RMSE) and mean absolute error (MAE) in addition to the previously reported (R2) scores. These complementary metrics provide a more complete assessment of predictive performance and facilitate interpretation of the quantitative results presented in the manuscript.

We are pleased that the reviewer finds the revised manuscript substantially improved and that the inclusion of the what-if analysis increases the value of the study.

Changes to the manuscript:

- We have added RMSE and MAE as error metrics for the SCM models in the Supplementary Information.
- We have expanded the method section concerning Shapley flows to improve understanding.

FURTHER CHANGES

Changes to the manuscript:

- We have updated all maps and added references to the sources of the respective shapefiles.
- To add the RMSE and MAE evaluation the SCM model was run through one more time leading to slight variation in LMC Violations due to randomness. This did not lead to an increase of LMC Violations.
